# FlareDB: A Database of Significant Flares in Solar Cycles 24 and 25 with SDO/HMI and SDO/AIA Observations

**DOI:** 10.1038/s41597-026-06607-7

**Published:** 2026-01-24

**Authors:** Nian Liu, Yasser Abduallah, Tanmay Sunil Kapure, Qin Li, Haimin Wang, Jason T. L. Wang

**Affiliations:** 1 Institute for Space Weather Sciences, New Jersey Institute of Technology, University Heights, Newark, NJ 07102-1982 USA; 2 Center for Solar-Terrestrial Research, New Jersey Institute of Technology, University Heights, Newark, NJ 07102-1982 USA; 3 College of Computing, New Jersey Institute of Technology, University Heights, Newark, NJ 07102-1982 USA

**Keywords:** Solar physics, Solar physics

## Abstract

We present FlareDB, a database that provides comprehensive magnetic field information, ultraviolet/extreme ultraviolet (UV/EUV) emissions, and white light continuum images for solar active regions (ARs) associated with 151 significant flares from May 2010 to May 2025. The data, sourced from the Solar Dynamics Observatory (SDO) via the Joint Science Operations Center (JSOC), were processed with SunPy and stored in standardized JSOC FITS format. FlareDB includes all M5.0 and larger flares within 50° of the solar disk center. Key features include (1) Atmospheric Imaging Assembly (AIA) AR patches in Helioprojective Cartesian(HPC) and Lambert Cylindrical Equal-Area (CEA) projections, aligned with corresponding HMI magnetogram patches; (2) quick-look movies with uniform value ranges that ensure consistent visualization, maintain data uniformity, and enhance readiness for machine learning studies; (3) a supplementary web interface that allows the entire dataset of a flare to be downloaded for large flare analysis. One of FlareDB’s primary objectives is to support scientists in predicting and understanding the onset of solar eruptions, including flares and coronal mass ejections. The data set is machine-learning ready for this purpose.

## Background & Summary

Solar flares are the Sun’s most intense energy-release events, involving restructuring of solar magnetic fields. Large flares, particularly those accompanied by coronal mass ejections (CMEs), can significantly impact space weather through their propagation in the interplanetary space^1^ and interactions with Earth’s magnetosphere^2–4^. They can also generate energetic particles and produce intense emissions across multiple wavelengths. Some of the events can disrupt Earth-based systems, including electric power grids^5^, aircraft and satellite operations, as well as telecommunications and navigation networks. Unfortunately, predicting space weather events including flares and CMEs that originate from the solar surface remains challenging because of the limited understanding of the initiation mechanisms^6–15^ of the eruptive events, particularly concerning the timing and scale of flares/CMEs.

Flare-related research faces two main challenges in advancing space weather forecasting. First, the most important group of events includes large flares, such as high M- and X-class flares. However, the availability of these samples is limited, which poses a challenge for statistical studies^16–20^ that require a robust dataset to predict such events. The diversity of flare classes across various ARs complicates systematic analysis, such as a single flare-producing AR can generate both small and large flares, mixing different levels of magnetic complexity and potentially blurring the data, which makes systematic analysis more challenging^21,22^. In addition, solar data analysis relies on multi-wavelength observations from different instruments^23^, which can be challenging to collect and organize due to differences in spatial and temporal resolution, data formats, calibration methods, and pointing accuracy between instruments. Due to the lack of a comprehensive dataset for flare-producing active regions, researchers often spend significant time conducting case-by-case studies, including visualizing multi-instrument images and tracking their temporal variations.

Second, flare-related research often faces limitations due to the lack of integration with modern data processing tools^24^. Machine learning (ML) and deep learning (DL), branches of artificial intelligence that mimic human learning to analyze data, offer promising solutions to handle large volumes of data in space weather studies^19,25–27^. For instance, DL models have demonstrated their capabilities in enhancing flare data analysis through image processing, such as predicting horizontal magnetic fields based on vertical magnetic field input, as demonstrated by Jiang *et al*.^28^ with an accuracy approaching 90% of real observations. Such advancements underscore the power of DL to extract valuable insights from complex, high-dimensional datasets. However, these tools can only reach their full potential when applied to well-curated datasets.

FlareDB is designed to address these two challenges by providing a robust, well-curated dataset of flare-producing ARs; it is a centralized repository that aims to enable efficient examination and standardization of data for flare-related research. Specifically, FlareDB contains white-light continuum images, magnetic field, and ultraviolet (UV) emission observations collected from two instruments on the Solar Dynamics Observatory (SDO)^29^. Magnetic field data, crucial for studying flare initiation mechanisms such as magnetic reconnection and magnetohydrodynamic (MHD) instabilities, are obtained from the Helioseismic and Magnetic Imager (HMI)^30^ on board SDO. UV data in two passbands (1600Å, 1700Å) and EUV data in seven passbands (94Å, 131Å, 171Å, 193Å, 211Å, 304Å, 335Å) are provided by the Atmospheric Imaging Assembly (AIA)^31^, which captures plasma distributions at temperatures ranging from several thousand to more than 20 million Kelvin in the solar atmosphere.

**Fig. 1:**
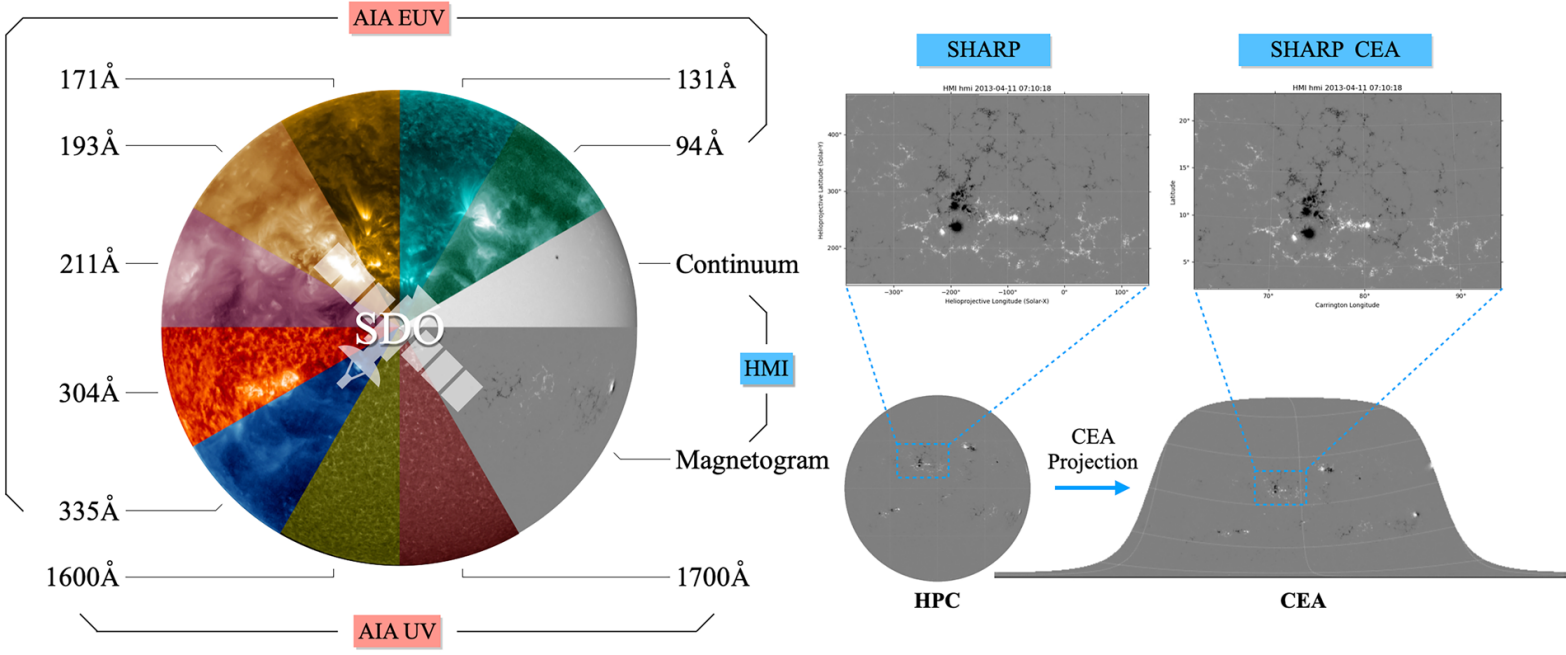
Overview of the data in FlareDB. The left panel shows multi-wavelength data obtained from SDO. The right panel displays HMI AR patches cropped from a full-disk magnetogram, shown in HPC and CEA coordinates respectively.

These data are categorized on the basis of two coordinate systems, as described in the hmi.sharp and hmi.sharp_cea series^32^ at the Joint Science Operations Center (JSOC), where hmi.sharp contains data in Helioprojective Cartesian (HPC)^33^ coordinates while hmi.sharp_cea contains data of the Lambert Cylindrical Equal-Area (CEA)^32^ projections in Carrington coordinates. Figure 1 presents an overview of the data.

The establishment of FlareDB enhances data analysis capabilities and modeling applications for both magnetic field information and UV/EUV emissions. For instance, the line-of-sight (LOS) magnetic field data provide fundamental structural insight into ARs and have been consistently observed by HMI over the past two solar cycles. Its high resolution vector magnetic field observations (0.5^*″*^ per pixel) with continuous 24-hour coverage have been available since May 2010. Prior to SDO, full disk LOS magnetograms had been observed by other instruments since mid-1970s, with the most recent one being MDI^34^, which was the predecessor of HMI. Additionally, systematical vector magnetic field data have been provided by VSM^35^/SOLIS^36^ since 2003.

Magnetic field analysis includes studies of AR topology evolution, sunspot dynamics, and the relationship between photospheric magnetic changes and CME/ICME coupling, along with a variety of magnetic field parameter calculations. The photospheric magnetic field is also widely used for reconstructing the coronal magnetic field. LOS magnetic field data can be used to extrapolate potential fields (PF) in 3D, while more complex methods, such as nonlinear force-free field (NLFFF)^37–39^ and non-force-free field (NFFF)^40–43^ extrapolations, require consistent vector magnetic field data.

Unlike magnetic field information, which typically varies over minutes or even hours, UV/EUV emissions change rapidly, with an observational cadence as short as 12 seconds—crucial for capturing the rapid dynamics of flares. This short response time allows UV/EUV observations to complement magnetograms, serving as a vital tool for validating magnetic-field structures derived from HMI data and tracking the rapid energy release processes during flares. Moreover, UV/EUV emissions carry crucial thermal and particle acceleration information during flare events, providing insight into the heating and energy transport mechanisms that characterize flare events. Recent applications, such as differential emission measure (DEM) analysis^44^, which assesses EUV emissions at different temperatures, and simulations that extrapolate EUV distributions within specific 3D volumes, have further highlighted the importance of EUV data in flare studies.

In summary, FlareDB, built using data from two SDO instruments (HMI and AIA), provides comprehensive resources for flare-related research with multi-wavelength and multi-cadence download options. The database can be used in four ways. (1) For case studies, all wavelengths have a uniform field of view and consistent image dimensions, focusing on the active region associated with a flare. (2) For statistical studies, a standardized data schema is used, with consistent data coverage duration and alignment around flare start times. (3) For image analysis, all solar images are compiled into quick look movies with a uniform value range, making them suitable for visualization applications. (4) For machine learning, the solar images in FlareDB can be used to train a deep learning model to predict the flare index of an AR^23^.

## Methods

A total of 151 significant (M5.0 and above) flares from 82 ARs were selected. This is a complete data set of events that satisfy certain criteria, detailed below. For each flare/AR, relevant solar images were downloaded from JSOC and processed using the Python-based library, SunPy^45^. Instead of providing full-disk images that require large storage, FlareDB focuses on observations within each selected AR. The download image size/dimension for each flare event is varied depending on the size of its AR. Details of our data processing methods are discussed in the following subsections.

### Selecting flare events

Significant flares and their ARs are selected according to the following criteria.

- The start time for each selected flare is in the range between 2010 and 2025.

**Fig. 2:**
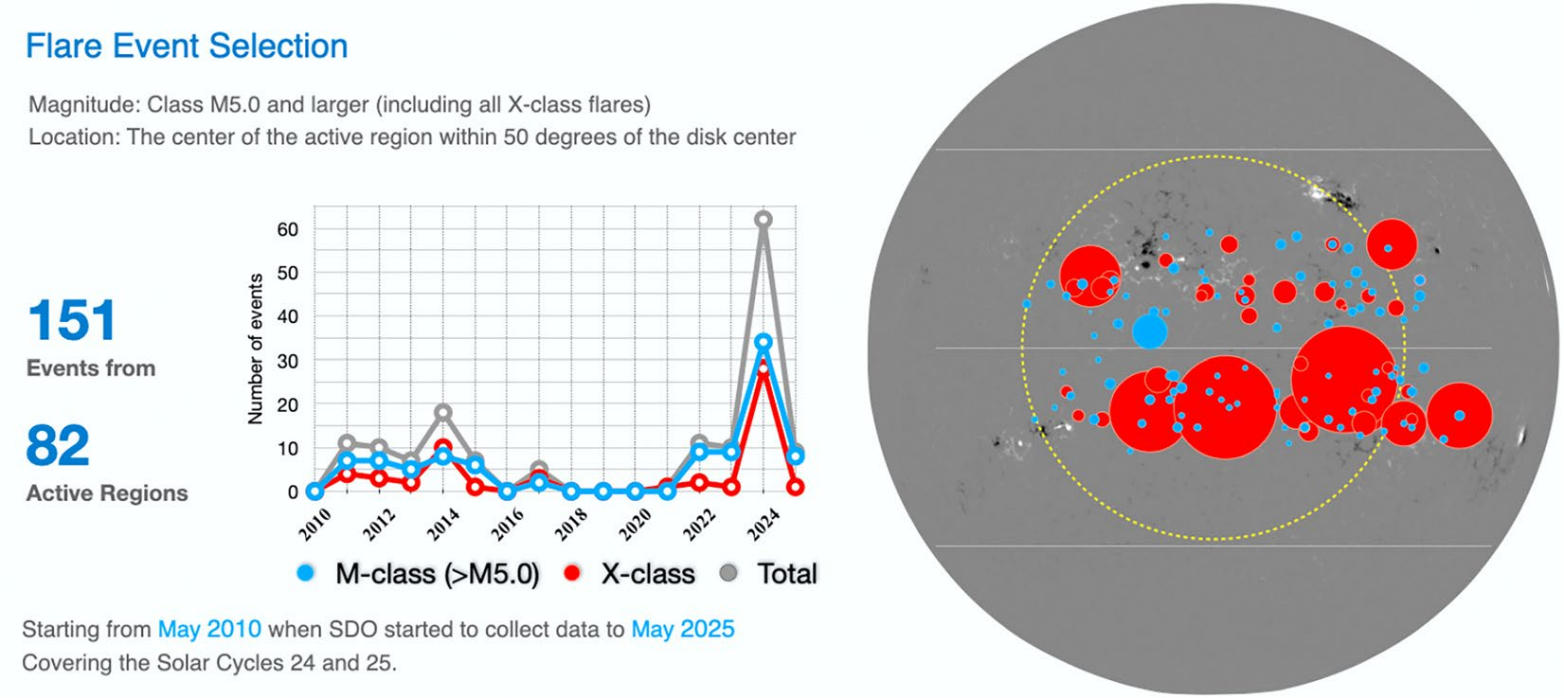
Statistics of the temporal and spatial distributions of the 151 significant flares in FlareDB. The spots on the full-disk magnetogram indicate the center locations of the ARs at the onsets of the flares. Blue and red spots represent large M-class (≥ M5.0) flares and X-class flares, respectively, with spot size indicating flare magnitude. The yellow dashed circle marks the selection limit of 50^°^ relative to the disk center.

SDO/HMI began operations in May 2010, providing systematic observations of full-disk vector magnetic field data that were not available from its predecessor, MDI. The selection period for the significant flares in FlareDB spans the major phase of the solar maximum in Solar Cycle 24, a period when the Sun is more active, which makes space weather prediction particularly important. It also includes the first six years of Solar Cycle 25, during which solar activity gradually increases starting from 2020. Figure 2 (left panel) shows the annual variation in the number of significant flares selected each year, reflecting the overall pattern of solar activity over the past 16 years. Solar Cycle 24 reached its maximum in 2014, with 18 events selected for that year. The year 2024 has also been highly active, where over 30 X-class flares were selected into FlareDB. Notably, AR 13663 and AR 13664, which generated super-geomagnetic storms in May 2024, are included in FlareDB.

- Each selected flare belongs to the M5.0 or higher class according to the NOAA soft X-ray (SXR) classification^46,47^.

FlareDB only includes X-class flares and large M-class (≥M5.0) flares, which can have significant impacts on space weather. The strongest flare in the database is an X9.3 flare that occurred on 6 September 2017, accompanied by a halo CME that released approximately 2.4 × 10^33^*erg* of kinetic energy. Flare events are used as the primary categorization unit in the database. Some ARs produce multiple qualified flares with very short time separations; each flare is treated as an individual event and stored in a separate database entry. For example, the X5.4 and X1.3 flares on 7 March 2012 occurred within two consecutive hours; they are stored separately in FlareDB, despite overlapping image files between the two events.

- The center of each AR which produces a qualified flare is located within 50^°^ of the solar disk center.

Applying this selection criterion excludes flares and the corresponding ARs near the solar limb for the following reasons. First, magnetic field observations near the limb are subject to larger uncertainties. The LOS magnetogram near the limb can differ significantly from that near the disk center. For example, the LOS direction for an AR at the solar center aligns closely with the Sun’s radial direction, while for an AR near the limb, the LOS direction is closer to the transverse field. Second, the Cylindrical Equal-Area (CEA) magnetic field data have greater errors near the limb.

Figure 2 (right panel) illustrates the distribution of AR locations at the onset of each corresponding flare. Most flare events in FlareDB are within the 50^°^ limit, marked by a yellow dashed circle. Some flares are beyond the right (western) side of this boundary. These flares are included because the dataset for each event begins 24 hours before the flare start time. Since active regions rotate approximately 13^°^ per day from the solar east to west, these flares still have substantial coverage of quality data within the 24-hour pre-flare window, warranting their inclusion.

### Downloading and preprocessing data

Multi-instrument data was downloaded from JSOC, which included the following types of data. For each flare/AR, the flare start time (*T*_0_) is set as a reference to determine the time coverage for each type of data. For all HMI data, as well as AIA data with a cadence of 12 minutes, the coverage time for each event starts 24 hours before and ends 8 hours after *T*_0_. For AIA data with a full cadence (12 seconds or 24 seconds), the time coverage for each event starts 5 minutes before *T*_0_ and ends 5 minutes after the flare end time (*T*_*end*_).

#### HMI data

We downloaded HMI AR patches in 12 minutes from the hmi.sharp_720s series at JSOC. The downloaded segments include “inclination”, “azimuth”, and “field”, representing inclination (*θ*) relative to the LOS, azimuth (*ϕ*) with zero degrees aligned to the north, and magnetic flux density (**B**), respectively, as well as “magnetogram”, and “continuum”, representing LOS magnetogram and continuum intensity, respectively. In addition, we downloaded HMI AR patches in CEA coordinates with a cadence of 12 minutes from the hmi.sharp_cea_720s series at JSOC. This series contains the same AR patches as the hmi.sharp_720s series, but with the vector magnetic field remapped to a Lambert Cylindrical Equal-Area (CEA) projection. Here, the magnetic field is decomposed into three components *B*_*p*_, *B*_*t*_, and *B*_*r*_, which correspond to the westward, southward, and radial (outward from the photosphere) components of the CEA vector magnetic field, respectively. The CEA data also include the LOS magnetogram, labeled “magnetogram”, and continuum intensity, labeled “continuum,” both of which are projected in the CEA format.

#### AIA data

We downloaded AIA UV full-disk images in HPC coordinates with a cadence of 24 seconds from the aia.lev1_uv_24s series at JSOC. The full-disk images are in 2 wavelengths: 1600Å and 1700Å. We also downloaded AIA UV full-disk images in HPC coordinates with a cadence of 12 minutes, where the AIA UV full-disk images temporally match the corresponding HMI AR patches with a cadence of 12 minutes downloaded previously. In addition, we downloaded AIA EUV full-disk images in HPC coordinates with a cadence of 12 seconds from the aia.lev1_euv_12s series at JSOC. The full-disk images are in 7 wavelengths: 94Å, 131Å, 171Å, 193Å, 211Å, 304Å, and 335Å. We also downloaded AIA EUV full-disk images in HPC coordinates with a cadence of 12 minutes where the AIA EUV full-disk images temporally match the corresponding HMI AR patches with a cadence of 12 minutes downloaded previously.

#### Data preprocessing

The downloaded HPC/CEA vector magnetograms are standardized in a uniform format with three components: *B*_*x*_, *B*_*y*_, and *B*_*z*_. For the HMI HPC data set, which includes magnetic flux density **B**, magnetic inclination *θ*, and azimuth *ϕ*, the vector components are calculated using the following equations:

1 $${B}_{x}={\bf{B}}\times sin(\theta )\times sin(\phi )$$

2 $${B}_{y}={\bf{B}}\times sin(\theta )\times cos(\phi )$$

3 $${B}_{z}={\bf{B}}\times cos(\theta )$$

One notable aspect related to the uncertainties of the vector components is that, in addition to the formal errors from instrumental and inversion limitations, the disambiguation process is another major source of uncertainty. The 180^°^ ambiguity problem arises when determining the azimuthal angle (*ϕ*) of the transverse field. Because of the symmetry of the Zeeman effect, there are two possible azimuthal directions that are indistinguishable and separated by 180^°^. The standard minimum energy algorithm (ME0^48,49^) is typically used to resolve this ambiguity and determine the reported azimuth. However, additional uncertainty is introduced when two alternative methods are used in cases where the main algorithm is inconclusive: the radial acute method and the random method^50^. The radial acute method selects the solution with the smaller (acute) angle between the transverse magnetic field and the local radial direction, and is applied in weak field regions or regions where the main algorithm lacks confidence. The random method is used in very weak fields or regions where neither the minimum energy algorithm nor the radial acute method can confidently resolve the ambiguity. In these cases, one of the two possible azimuthal directions is chosen at random.

For the HMI CEA vector magnetograms, since their components *B*_*p*_, *B*_*t*_, and *B*_*r*_ are already aligned with Cartesian coordinates, we apply the following simple conversion:

4 $${B}_{x}={B}_{p},{B}_{y}=-{B}_{t},{B}_{z}={B}_{r}$$

It is worth mentioning that the image sizes in each AR are standardized. Since the collected AR patches/images span 32 hours per event, the location of the AR shifts by approximately 17.3^°^ from the start to the end of each data sequence. As a result, the image dimensions in the AR vary slightly, particularly in the horizontal direction, by about 10 pixels. To address the size/dimension difference, a uniform image size is set before any calculation or alignment, with the AR consistently centered within each image.

#### Creating AIA AR patches in HPC and CEA coordinates

When AIA full-disk images are aligned with HMI AR magnetograms, resolution becomes a primary source of uncertainty, as the resolution for AIA images is 0.6^*″*^/pixel, while the resolution for HMI magnetograms is 0.5^*″*^/pixel. For AIA images in HPC coordinates, we retain the original AIA resolution, even though this results in a difference in the image size for the same field of view (FOV) between AIA and HMI. We align AIA HPC full-disk images with the corresponding HMI HPC AR patches that temporally match the AIA images and crop the AIA full-disk images into AR patches in HPC coordinates. However, for AIA images in CEA coordinates, the full-disk AIA images need to be projected onto a square FOV with a manually set resolution. In this case, we apply the same resolution, namely 0.5^*″*^/pixel, to AIA and HMI images to ensure uniform final image sizes.

**Fig. 3:**
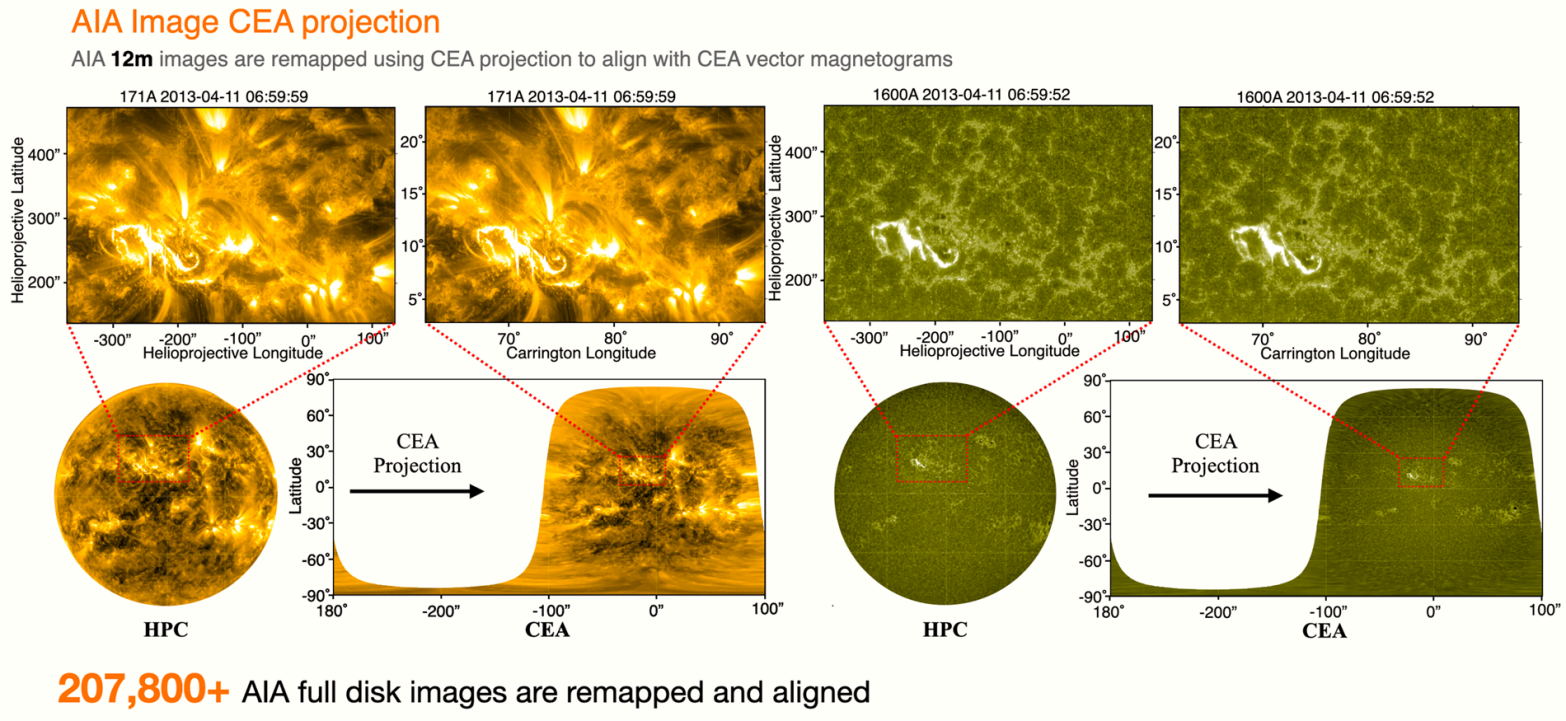
AIA full-disk images in 171Å and 1600Å remapped from HPC to CEA coordinates using CEA projection. AIA HPC AR patches are cropped from AIA HPC full-disk images and aligned with HMI HPC AR patches. AIA CEA AR patches are cropped from AIA CEA full-disk images and aligned with HMI CEA AR patches.

Figure 3 shows examples of full-disk AIA HPC image remapped into cylindrical equal-area projections, represented as squares with dimensions of 360^°^ in longitude and 180^°^ in latitude. Notably, unlike photospheric magnetograms, where values represent a thin, near-2D surface layer of the photosphere, some AIA wavelength images capture emissions from a volume with significant thickness. This distinction is particularly evident for EUV wavelengths at 94Å, 193Å, 211Å, and 335Å, as these channels capture emissions from the full coronal volume. Consequently, careful consideration is required when processing AIA images in these wavelengths. Furthermore, the CEA projections for AIA full-disk images are applied only to 12-minute cadence data because each alignment requires reference coordinate information from the corresponding HMI CEA magnetogram, which is available every 12 minutes. During flaring periods, the variations in AIA images are significantly more intense than those in HMI magnetograms, making the direct correspondence between AIA data (with a cadence of 12 seconds) and HMI data (with a cadence of 12 minutes) less meaningful.

In summary, more than 218,000 AIA full-disk images are remapped from HPC to CEA coordinates using CEA projection, then aligned with the corresponding HMI CEA AR patches and cropped into AIA CEA AR patches. This is one of the key innovations of FlareDB, as the database provides comprehensive AIA HPC/CEA AR images that are aligned precisely with corresponding HMI HPC/CEA AR magnetograms.

#### Producing quick look movies

**Fig. 4:**
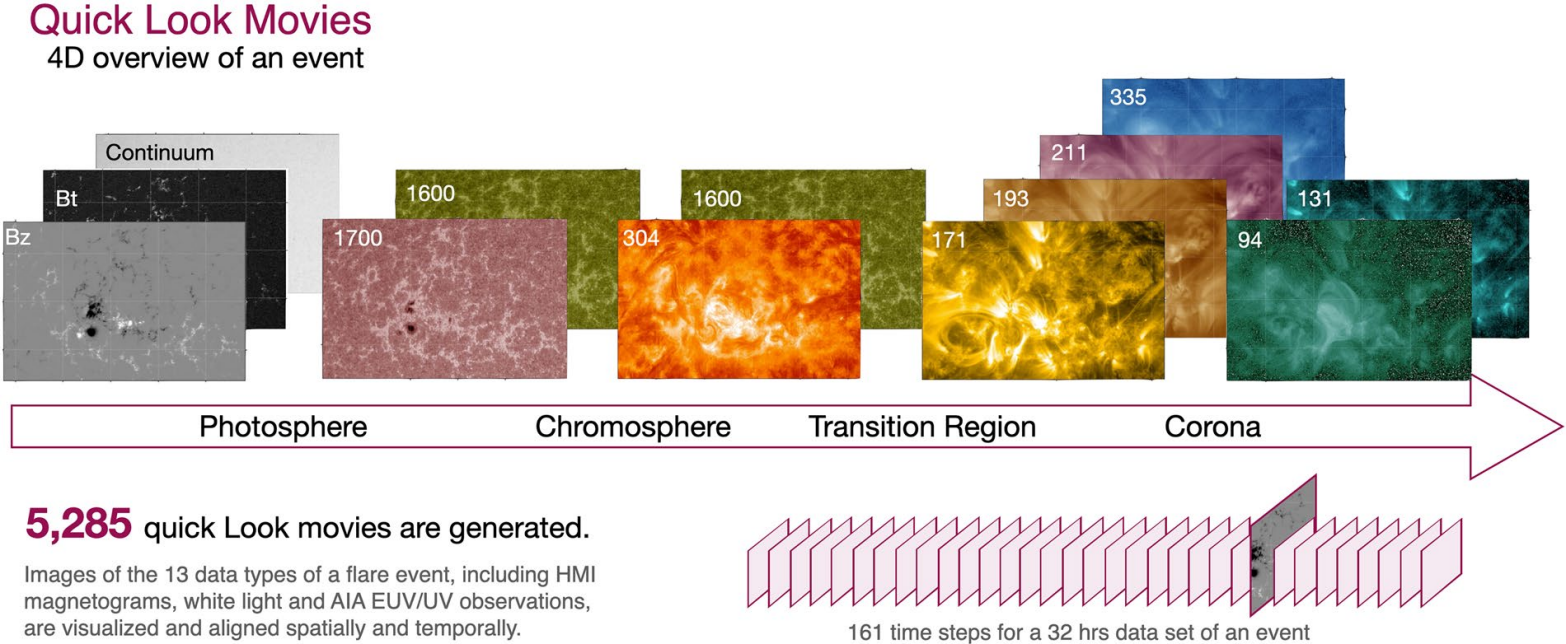
Overview of the quick look movie for each data type and the corresponding atmosphere/structure from the photosphere to corona for a flare event. Displayed in the figure is an example image for each data type at a randomly selected time point for the event.

For each flare event, 35 quick look movies are generated, showing HMI and AIA EUV/UV observations in both HPC and CEA coordinates. These movies provide a four-dimensional overview of each event by presenting time series of two-dimensional (2D) images across multiple wavelengths, capturing different layers from the photosphere to the corona. The quick look movies for AIA data use a standard color table for each individual wavelength. The magnetograms are plotted with a gray-scale color table. Figure 4 shows an example image for each data type at a randomly selected time point for an event with multi-wavelength and magnetic field information. For each event, there are 161 time points/steps that span 32 hours (that is, 24 hours before and 8 hours after the start time of the event). We used HMI data as the main structure, pairing AIA data with each corresponding HMI image that temporally matches the AIA data. Unlike other data types, where a movie is produced for a single wavelength or data type, the movie for the data type *B*_*t*_ illustrates the transverse field, which combines both data types, *B*_*x*_ and *B*_*y*_, according to the relation: ${B}_{t}=\sqrt{{B}_{x}^{2}+{B}_{y}^{2}}$. To ensure consistency, all quick look movies are produced with a uniform value range. For HMI data, the magnetic flux density of a pixel ranges from  −1000 to 1000 Gauss for *B*_*z*_ and from 0 to 1500 Gauss for *B*_*t*_. Continuum movies have a value range of 10,000 to 50,000 DN/s. For AIA images, the value ranges vary by wavelength. In total, (151 × 35) = 5,285 quick look movies are produced (see Fig. 4), standardized in a uniform value range as shown in Table 1.

**Table 1:** Value range for each data type used in producing quick look movies. footnote Totally, there are 13 data types in FlareDB.

Data Type (Unit)	Minimum	Maximum	Data Type (Unit)	Minimum	Maximum
Magnetogram (Gauss)	− 1, 000	1,000	94Å, 304Å (DN/s)	1	1,000
Continuum (DN/s)	10,000	50,000	131Å (DN/s)	5	2,000
Bz (Gauss)	− 1, 000	1,000	171Å (DN/s)	50	3,000
Bt (Gauss)	0	1,500	193Å (DN/s)	100	10,000
1600Å (DN/s)	50	2,000	211Å (DN/s)	100	5,000
1700Å (DN/s)	200	8,000	335Å (DN/s)	1	900

## Data Records

The dataset is publicly available on Zenodo^51^. The quick-look movies, which constitute the essential part of FlareDB, are standardized using consistent value ranges (see Table 1). In total, 5,285 movies corresponding to 151 significant flare events from 82 active regions are publicly available for download from the permanent repository. They are stored in 35 compressed files, with each file containing 151 movies corresponding to 151 events of a specific data type.

**Fig. 5:**
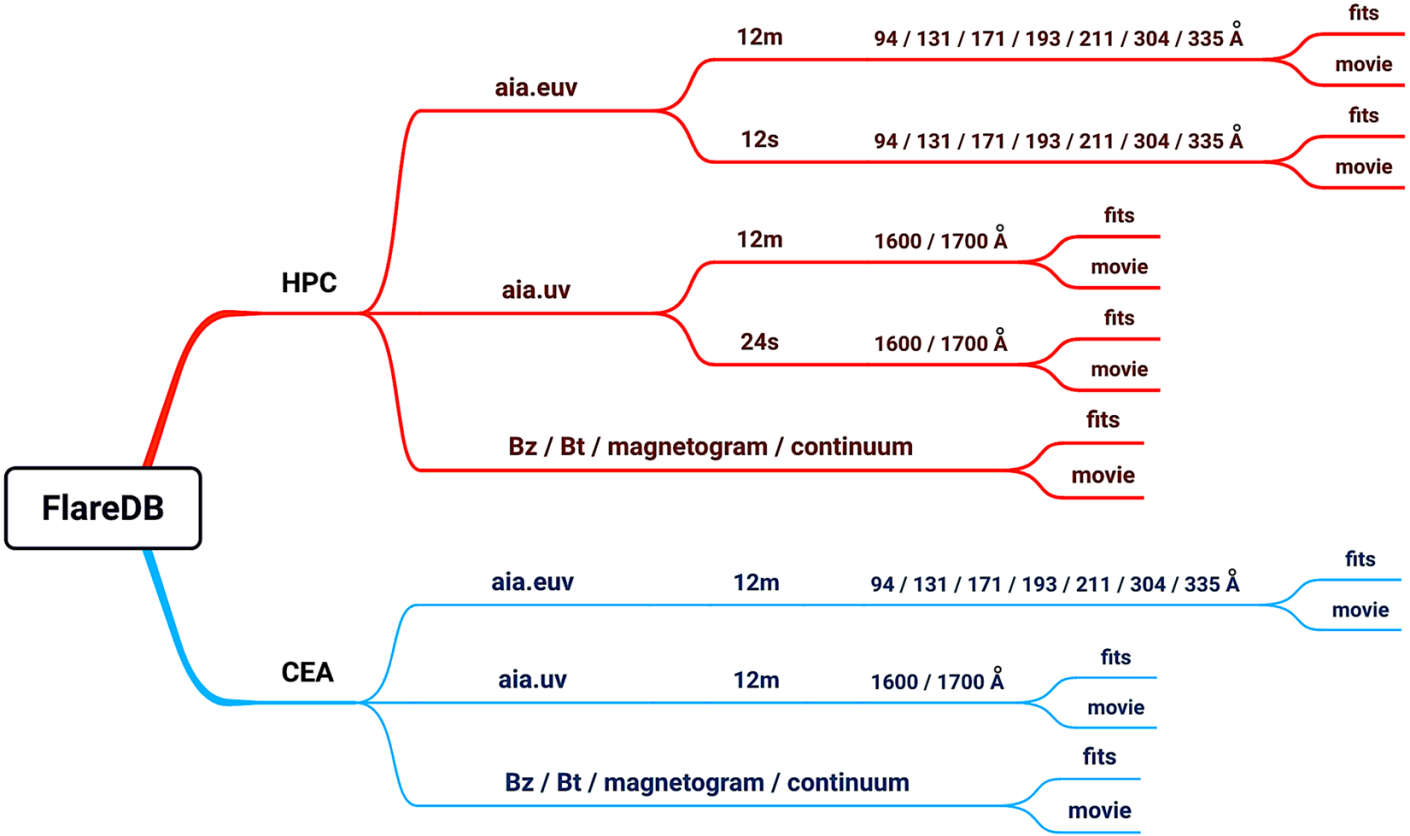
The data organization chart of FlareDB. This chart illustrates how the data is organized for each flare event, which guides the user to browse all the data associated with the event.

The files are named according to the format: “AIA_coordinates_cadence_wavelength.zip” for AIA data and “HMI_coordinates_Bz/Bt/continuum/magnetogram.zip” for HMI data. Note that for the HPC data, the high-cadence (12-second or 24-second) AIA movies cover only the flare time and are not available for the CEA data. In addition, two supplementary files are included in the dataset: one illustrates the FlareDB organization chart (same as Fig. 5), and the other provides the flare event list, which includes the soft X-ray class, NOAA active region number, flare start, peak, and end times, as well as the HMI Active Region Patches (HARP) number for each event. Each movie is named after the active region number, HARP number, and flare start time. Furthermore, each movie is named according to the active region number, HARP number, and flare start time, making it easy to locate the movie.

**Fig. 6:**
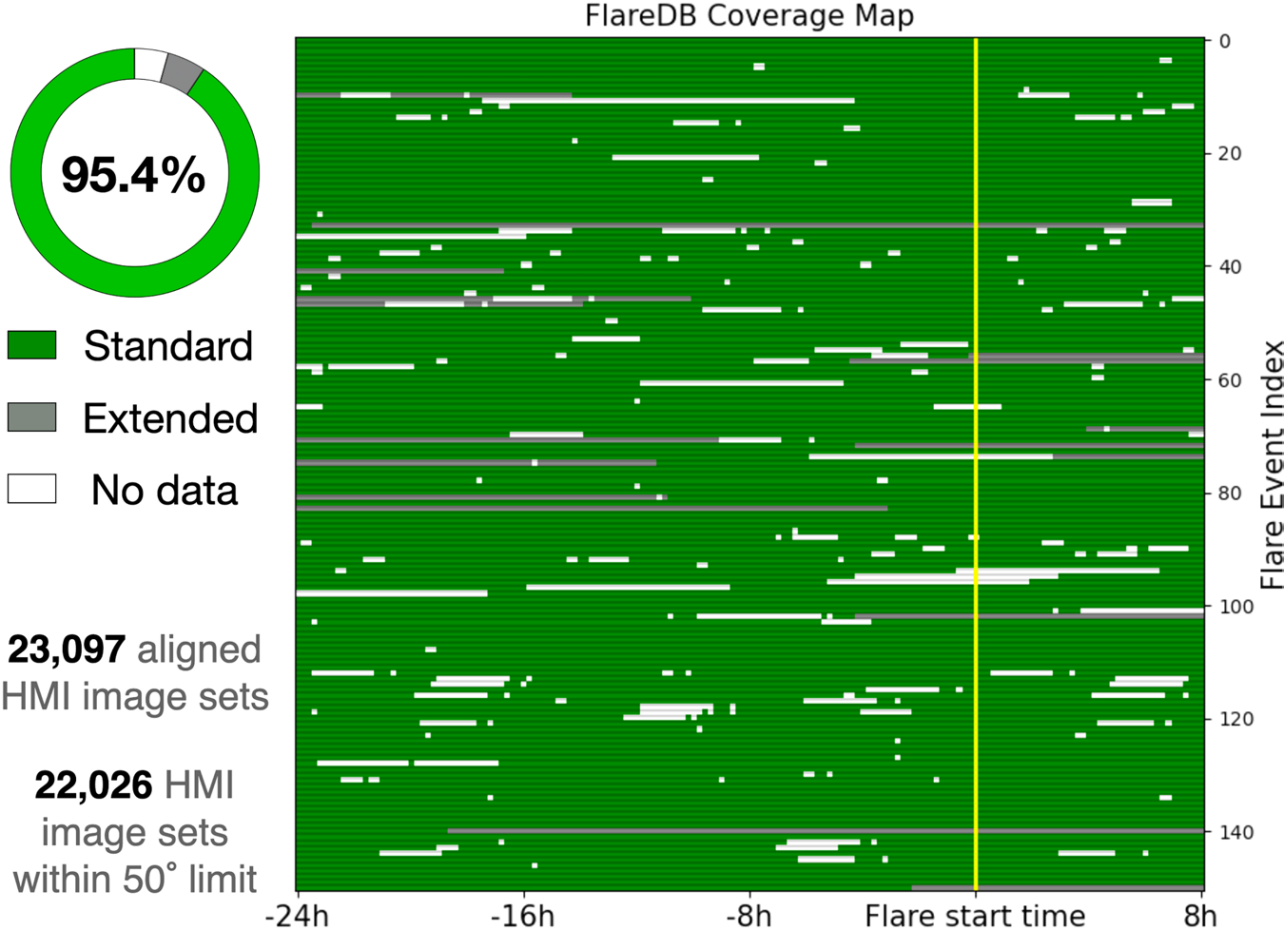
FlareDB data coverage map of 151 flare events. The standard data and extended data correspond to time slots where the image center is located within or outside 50^°^ of the solar disk center, respectively. The proportion of standard data accounts for 95.4% of all available data (standard and extended).

The completeness of FlareDB is illustrated in Fig. 6. In this figure, green and gray areas indicate time slots when the full dataset including all data types (HMI and 12-minute AIA) is available. The difference between them is that the green areas correspond to events whose image centers are located within 50^°^ of the solar disk center, which we refer to as the standard data. As explained in the subsection Selecting Flare Events and shown in Fig. 2, some events that partially exceed the 50^°^ limit are also included. The portions of these events beyond the 50^°^ limit are marked as gray in Fig. 6, which we refer to as the extended data.

## Technical Validation

The reliability of FlareDB data is based on the quality of JSOC data. Therefore, our technical validation focuses on how the processing of the original JSOC data affects their quality. The HPC and CEA products are generated separately, but both follow the same processing sequence: vector magnetogram calculation, AIA image alignment and cropping, and finally, movie generation. The latter two steps depend on the quality of the magnetogram production.

For the HPC product line, the main difference between the FlareDB magnetograms and the SHARP magnetograms lies in boundary adjustments. As explained in the Data Preprocessing subsection, the dimensions of the LOS magnetograms vary over the 32-hour coverage. FlareDB standardizes these dimensions by selecting the smallest field of view (FOV) as the uniform size and consistently centering the active region in each image. During this process, trimming larger images may result in a loss of data. To limit this effect, we applied a threshold: if the averaged maximum magnetic flux density lost during cropping exceeds 50 Gauss, the trimming is considered unacceptable.

We intentionally retain both “Bz” and “magnetogram” data types in the HPC and CEA products, as recognizing their differences is important for proper use. For HPC data, the physical meaning of “Bz” and “magnetogram” is the same, since both represent the LOS magnetogram. The distinction lies in their uncertainties: “magnetogram” is taken directly from the hmi.M_720s series, while “Bz” is derived from the hmi.B_720s series. Because the disambiguation procedure introduces additional uncertainty, the “magnetogram” exhibits smaller uncertainties. For CEA data, however, their physical meanings are different. In this case, “Bz” represents the radial component of the flux density, while “magnetogram” corresponds to the LOS field remapped into CEA coordinates without projection. In other words, the CEA “magnetogram” still reflects the LOS component, rather than a true radial field. This direct remapping of the magnetogram also correlates more naturally with the AIA CEA products, since EUV emissions are LOS intensity, making the CEA magnetogram a better complementary non-vector value for EUV analysis.

For events that occur within the same active region, the 32-hour data is collected on an event-based scheme. The active regions and events are not in a one-to-one correspondence. As a result, the data coverage for two events may overlap if they are not separated temporally by 32 hours. In addition, Fig. 6 shows that, for some events, a significant portion of the 32-hour coverage extends beyond 50^°^ from the solar disk center. In contrast, events from different active regions generally differ in their FOV. Therefore, the temporal and spatial coverage for each event should be balanced according to the specific purpose of the study.

## Usage Notes

For many events, the actual maximum values of each data type may significantly exceed those displayed in the movies. The advantage of these movies lies in their uniform data value ranges across all flare events, with each movie manually validated. However, this uniformity comes with a trade-off that some details may be lost because the fixed ranges cannot fully capture the varying intensities of different flare events. If users require single image files, we provide full access to the original FITS files through SolarDB, a cyberinfrastructure designed to advance space weather research, as a supplementary data source. These FITS files can be downloaded following the data organization chart shown in Fig. 5.

To access FlareDB on SolarDB, visit https://nature.njit.edu/solardb/. From the top menu, click Databases, then select Flare Database from the drop-down menu. This leads to the main page of FlareDB, where users can search for flares by date/time, NOAA AR number, and flare class. Each FITS file contains a 2D image along with metadata describing its properties. Files can be downloaded individually or as compressed TAR archives, which include all files of a given type for a single event. As a recommended workflow, users may first view the quick-look movies to decide whether to download the corresponding FITS files. Please download the TAR archives with care and patience considering the network bandwidth and the capacity of the FlareDB host server. https://github.com/Reasopprime/njit-flaredb/.

## Data Availability

The dataset is publicly available at the Zenodo repository: 10.5281/zenodo.16790538^51^.

## Code availability

The data processing scripts, including data downloading, completeness checks, cropping and alignment of images, and movie creation, are available in the github repository: https://github.com/Reasopprime/njit-flaredb/.

## References

[1] Gonzalez, W. D., Tsurutani, B. T. & Clúa de Gonzalez, A. L. Interplanetary origin of geomagnetic storms. *Space Science Reviews* **88**, 529–562, 10.1023/A:1005160129098 (1999).

[2] Schrijver, C. J. & Siscoe, G. L., *Heliophysics: Evolving Solar Activity and the Climates of Space and Earth*. Cambridge University Press, Cambridge, UK. (2010).

[3] Vemareddy, P. & Mishra, W. A full study on the Sun-Earth connection of an Earth-directed CME magnetic flux rope. *The Astrophysical Journal* **814**(1), 59, 10.1088/0004-637X/814/1/59 (2015).

[4] Temmer, M. *et al*. Flare-CME characteristics from Sun to Earth combining observations and modeling. In. *EGU General Assembly Conference Abstracts* **19**, 1942 (2017).

[5] Pulkkinen, A., Bernabeu, E., Eichner, J., Viljanen, A. & Ngwira, C. Regional-scale high-latitude extreme geoelectric fields pertaining to geomagnetically induced currents. *Earth, Planets and Space* **67**, 93, 10.1186/s40623-015-0255-6 (2015).

[6] Bateman, G., *MHD Instabilities*. OSTI.GOV, United States. https://www.osti.gov/biblio/6503774 (1978).

[7] Amari, T., Luciani, J. F., Aly, J. J. & Tagger, M. Very fast opening of a three-dimensional twisted magnetic flux tube. *The Astrophysical Journal* **466**(1), L39–L42, 10.1086/310158 (1996).

[8] Antiochos, S. K., DeVore, C. R. & Klimchuk, J. A. A model for solar coronal mass ejections. *The Astrophysical Journal* **510**(1), 485–493, 10.1086/306563 (1999).

[9] Moore, R. L., Sterling, A. C., Hudson, H. S. & Lemen, J. R. Onset of the magnetic explosion in solar flares and coronal mass ejections. *The Astrophysical Journal* **552**(2), 833–848, 10.1086/320559 (2001).

[10] Tokman, M. & Bellan, P. M. Three-dimensional model of the structure and evolution of coronal mass ejections. *The Astrophysical Journal* **567**(2), 1202–1210, 10.1086/338699 (2002).

[11] MacNeice, P. *et al*. A numerical study of the breakout model for coronal mass ejection initiation. *The Astrophysical Journal* **614**(2), 1028–1041, 10.1086/423887 (2004).

[12] Török, T., Kliem, B. & Titov, V. S. Ideal kink instability of a magnetic loop equilibrium. *Astronomy & Astrophysics* **413**(3), L27–L30, 10.1051/0004-6361:20031691 (2004).

[13] Kliem, B. & Török, T. Torus instability. *Physical Review Letters* **96**(25), 255002, 10.1103/physrevlett.96.255002 (2006). 16907312 10.1103/PhysRevLett.96.255002

[14] Ishiguro, N. & Kusano, K. Double arc instability in the solar corona. *The Astrophysical Journal* **843**(2), 101, 10.3847/1538-4357/aa799b (2017).

[15] Green, L. M., Török, T., Vršnak, B., Manchester, W. & Veronig, A. The origin, early evolution and predictability of solar eruptions. *Space Science Reviews* **214**(1), 46, 10.1007/s11214-017-0462-5 (2018).

[16] Georgoulis, M. K. & Rust, D. M. Quantitative forecasting of major solar flares. *The Astrophysical Journal Letters* **661**(1), L109–L112, 10.1086/518718 (2007).

[17] Gopalswamy, N., Xie, H., Akiyama, S., Mäkelä, P. A. & Yashiro, S. Major solar eruptions and high-energy particle events during solar cycle 24. *Earth, Planets and Space* **66**, 104, 10.1186/1880-5981-66-104 (2014).

[18] Barczynski, K. *et al*. A statistical comparison of EUV brightenings observed by SO/EUI with simulated brightenings in nonpotential simulations. *Solar Physics* **297**(10), 141, 10.1007/s11207-022-02074-6 (2022). 36310545 10.1007/s11207-022-02074-6 PMC9606066

[19] Zhang, H. *et al*. Solar flare index prediction using SDO/HMI vector magnetic data products with statistical and machine-learning methods. *The Astrophysical Journal Supplement Series* **263**(2), 28, 10.3847/1538-4365/ac9b17 (2022).

[20] Karimov, K. *et al*. 3D magnetic free energy and flaring activity using 83 major solar flares. *The Astrophysical Journal Letters* **965**(1), L5, 10.3847/2041-8213/ad3548 (2024).

[21] Mackay, D. H. & Yeates, A. R. The Sun’s global photospheric and coronal magnetic fields: observations and models. *Living Reviews in Solar Physics* **9**(1), 6, 10.12942/lrsp-2012-6 (2012).

[22] Toriumi, S. & Wang, H. Flare-productive active regions. *Living Reviews in Solar Physics* **16**(1), 3, 10.1007/s41116-019-0019-7 (2019). 31178676 10.1007/s41116-019-0019-7 PMC6530820

[23] Liu, N., Jing, J., Xu, Y. & Wang, H. Multi-instrument comparative study of temperature, number density, and emission measure during the precursor phase of a solar flare. *The Astrophysical Journal* **930**(2), 154, 10.3847/1538-4357/ac6425 (2022).

[24] Camporeale, E. The challenge of machine learning in space weather: nowcasting and forecasting. *Space Weather* **17**(8), 1166–1207, 10.1029/2018SW002061 (2019).

[25] Liu, H., Liu, C., Wang, J. T. L. & Wang, H. Predicting solar flares using a long short-term memory network. *The Astrophysical Journal* **877**(2), 121, 10.3847/1538-4357/ab1b3c (2019).

[26] Liu, H., Liu, C., Wang, J. T. L. & Wang, H. Predicting coronal mass ejections using SDO/HMI vector magnetic data products and recurrent neural networks. *The Astrophysical Journal* **890**(1), 12, 10.3847/1538-4357/ab6850 (2020).

[27] Alobaid, K. A. *et al*. Estimating coronal mass ejection mass and kinetic energy by fusion of multiple deep-learning models. *The Astrophysical Journal Letters* **958**(2), L34, 10.3847/2041-8213/ad0c4a (2023).

[28] Jiang, H. *et al*. Generating photospheric vector magnetograms of solar active regions for SOHO/MDI using SDO/HMI and BBSO data with deep learning. *Solar Physics* **298**(7), 87, 10.1007/s11207-023-02180-z (2023).

[29] Pesnell, W. D., Thompson, B. J. & Chamberlin, P. C. The Solar Dynamics Observatory (SDO). *Solar Physics* **275**(1), 3–15, 10.1007/s11207-011-9841-3 (2012).

[30] Scherrer, P. H.*et al*. The Helioseismic and Magnetic Imager (HMI) investigation for the Solar Dynamics Observatory (SDO). In *The Solar Dynamics Observatory* (pp. 207–227). Springer US, New York, NY. 10.1007/978-1-4614-3673-7_10 (2012).

[31] Lemen, J. R. *et al*. The Atmospheric Imaging Assembly (AIA) on the Solar Dynamics Observatory (SDO). *Solar Physics* **275**(1), 17–40, 10.1007/s11207-011-9776-8 (2012).

[32] Bobra, M. G. *et al*. The Helioseismic and Magnetic Imager (HMI) vector magnetic field pipeline: SHARPs - Space-Weather HMI Active Region Patches. *Solar Physics* **289**(9), 3549–3578, 10.1007/s11207-014-0529-3 (2014). 10.1007/s11207-015-0686-z PMC4456067 26069350

[33] Thompson, W. T. Coordinate systems for solar image data. *Astronomy and Astrophysics* **449**(2), 791–803, 10.1051/0004-6361:20054262 (2006).

[34] Scherrer, P. H., Bogart, R. S. & Bush, R. I. The Solar Oscillations Investigation - Michelson Doppler Imager. *Solar Physics* **162**(1-2), 129–188, 10.1007/BF00733429 (1995).

[35] Bertello, L. & Marble, A. R. SOLIS/VSM polar magnetic field data. arXiv preprint arXiv:1507.07976. https://arxiv.org/abs/1507.07976 (2015).

[36] Keller, C. U., Harvey, J. W. & Giampapa, M. S., (2003). SOLIS: an innovative suite of synoptic solar instruments. In *Innovative Telescopes and Instrumentation for Solar Astrophysics*, edited by S. L. Keil & S. V. Avakyan, Vol. 4853, pp. 194-204. *International Society for Optics and Photonics (SPIE)*. 10.1117/12.460373 (2003).

[37] Wheatland, M. S., Sturrock, P. A. & Roumeliotis, G. An optimization approach to reconstructing force-free fields. *The Astrophysical Journal* **540**(2), 1150–1155, 10.1086/309355 (2000).

[38] Wiegelmann, T. Optimization code with weighting function for the reconstruction of coronal magnetic fields. *Solar Physics* **219**(1), 87–108, 10.1023/b:sola.0000021799.39465.36 (2004).

[39] Wiegelmann, T., Inhester, B. & Sakurai, T. Preprocessing of vector magnetograph data for a nonlinear force-free magnetic field reconstruction. *Solar Physics* **233**(2), 215–232, 10.1007/s11207-006-2092-z (2006).

[40] Hu, Q. & Dasgupta, B., (2006). A new approach to modeling non-force-free coronal magnetic field. *Geophysical Research Letters*, **33**1. 10.1029/2006GL026952

[41] Hu, Q. & Dasgupta, B. An improved approach to non-force-free coronal magnetic field extrapolation. *Solar Physics* **247**(1), 87–101, 10.1007/s11207-007-9090-7 (2008).

[42] Hu, Q., Dasgupta, B., Choudhary, D. P. & Büchner, J. A practical approach to coronal magnetic field extrapolation based on the principle of minimum dissipation rate. *The Astrophysical Journal* **679**(1), 848–853, 10.1086/587639 (2008).

[43] Prasad, A., Bhattacharyya, R. & Kumar, S. Magnetohydrodynamic modeling of solar coronal dynamics with an initial non-force-free magnetic field. *The Astrophysical Journal* **840**(1), 37, 10.3847/1538-4357/aa6c58 (2017).

[44] Cheung, M. C. M. *et al*. Thermal diagnostics with the Atmospheric Imaging Assembly on board the Solar Dynamics Observatory: a validated method for differential emission measure inversions. *The Astrophysical Journal* **807**(2), 143, 10.1088/0004-637X/807/2/143 (2015).

[45] Barnes, W. T. *et al*. The SunPy project: open source development and status of the version 1.0 core package. *The Astrophysical Journal* **890**(1), 68, 10.3847/1538-4357/ab4f7a (2020).

[46] Thomas, R. J., Starr, R. & Crannell, C. J. Expressions to determine temperatures and emission measures for solar X-ray events from GOES measurements. *Solar Physics* **95**(2), 323–329, 10.1007/BF00152409 (1985).

[47] White, S. M., Thomas, R. J. & Schwartz, R. A. Updated expressions for determining temperatures and emission measures from GOES soft X-ray measurements. *Solar Physics* **227**(2), 231–248, 10.1007/s11207-005-2445-z (2005).

[48] Metcalf, T. R. Resolving the 180-degree ambiguity in vector magnetic field measurements: the ’minimum’ energy solution. *Solar Physics* **155**(2), 235–242, 10.1007/BF00680593 (1994).

[49] Leka, K. D. *et al*. Resolving the 180° ambiguity in solar vector magnetic field data: evaluating the effects of noise, spatial resolution, and method assumptions. *Solar Physics* **260**(1), 83–108, 10.1007/s11207-009-9440-8 (2009).

[50] Hoeksema, J. T. *et al*. The Helioseismic and Magnetic Imager (HMI) vector magnetic field pipeline: overview and performance. *Solar Physics* **289**(9), 3483–3530, 10.1007/s11207-014-0516-8 (2014).

[51] Liu, N.*et al*. FlareDB: a database of significant flares in solar cycles 24 and 25 with SDO/HMI and SDO/AIA observations—quick-look movies. *Zenodo*, version 2025-05-31. 10.5281/zenodo.16790538 (2025). 10.1038/s41597-026-06607-7 PMC12920639 41580394

